# Effect of biological and agricultural foaming agents on concrete waste for the preparation of porous ceramic materials

**DOI:** 10.1038/s41598-026-52176-5

**Published:** 2026-05-19

**Authors:** R. M. Khattab, H. H. Abo-Almaged, Momen M. Ali, H. E. H. Sadek, M. A. Marzouk

**Affiliations:** 1 Refractories, Ceramics and Building Materials Department, National Research Centre (NRC), Dokki, Giza, 12622, 60014618 Egypt; 2 Construction Engineering and Management Department, Faculty of Engineering, Pharos University, Alexandria, Egypt; 3 Glass Research Department, National Research Centre, 33 EL Bohouth St. (former EL Tahrir St.), Dokki, P.O.12622, Giza, Egypt

**Keywords:** Porous ceramic, Thermal transmittance, Concrete waste, Wheat straw, Sawdust, Engineering, Materials science

## Abstract

This study offers a novel method for recycling leftover concrete to create porous ceramic materials. Various pouring agents, such as starch mixed with flour, yeast, baking powder, wheat straw, or sawdust, have created porous waste concrete ceramic materials. The starch is a binder for ceramic particles and a growth substrate for forming gas bubbles. Utilizing starch consolidation procedures, the green bodies are produced by firing them at temperatures of 1200 °C, 1250 °C, and 1300 °C. Various techniques characterize the fired sample, including Fourier Transform Infrared Spectroscopy, Thermogravimetric analysis, Differential Scanning Calorimetry, Apparent porosity, Bulk density, Compressive strength, Phase composition, and Microstructure. The acquired results showed that apparent porosity decreased except for samples containing baking powders, while the bulk density and compressive strength increased as the firing temperature increased. With apparent porosity of 56% and 39%, bulk density of 1.27 g/cm^3^ and 1.64 g/cm^3^, and mechanical characteristics of 0.55 MPa and 1.47 MPa at 1250 °C and 1300 °C, respectively, samples containing flour have a highly porous structure. At 1250 °C, the thermal transmittance of this sample reaches 0.327 W/m^2^K. The sawdust-containing sample exhibited the highest strength, reaching 1.54 MPa at 1250 °C and 5.23 MPa at 1300 °C. At those respective temperatures, its apparent porosity measured approximately 55% and 38%. Additionally, sawdust samples had a thermal transmittance of up to 0.336 W/m^2^K. Therefore, the current study created porous structure ceramics by substituting all or some of the more expensive polymer additives with recycled concrete debris. The resulting ceramics are potentially used in gas burners, membranes, filters, and refractory thermal conductivity.

## Introduction

The continuous and rapid expansion of urban infrastructure, driven by population growth, industrial development, and modernization, has resulted in a significant escalation in the demand for construction materials worldwide. This increase in construction activity has led to an exceptionally high annual consumption of natural aggregates, such as sand and gravel, which are fundamental components of concrete production. As a consequence, the extensive exploitation of these natural resources has reached a critical level, raising serious concerns regarding the progressive depletion and growing scarcity of natural aggregates^1–4^. Simultaneously, the demolition, renovation, and replacement of aging or obsolete structures generate enormous quantities of construction and demolition waste each year. Among the various types of waste produced, waste concrete constitutes the largest proportion, primarily due to its widespread use in modern construction practices. In many regions, the management of this waste remains inadequate, leading to the uncontrolled and untreated disposal of construction debris, particularly in suburban and peri-urban areas. Such disposal practices exert considerable pressure on land resources and result in severe degradation of the surrounding biological and ecological environments, including soil contamination, landscape deterioration, and potential risks to human health^1,4–8^.

In light of these challenges, the recycling and reutilization of residual concrete materials have become increasingly important. Recycling waste concrete not only reduces the demand for natural aggregates but also mitigates environmental pollution and decreases the volume of waste sent to landfills. Moreover, the adoption of concrete recycling strategies contributes to sustainable resource management, enhances environmental protection, and generates notable economic and societal benefits. Therefore, the effective recycling of leftover concrete plays a vital role in promoting sustainable development within the construction industry.

From this perspective, the development of porous ceramic materials using waste concrete emerges as a highly promising and sustainable strategy for addressing the environmental challenges associated with construction and demolition waste. Porous ceramics are defined as solid materials characterized by a network of pores, which can be isolated, interconnected, or permeated by fluids such as liquids or gases^9,10^. The distribution, size, and connectivity of these pores play a critical role in determining the physical, mechanical, and functional properties of the material^9–12^. For instance, open and interconnected pores facilitate the passage and storage of fluids, making such ceramics highly suitable for applications in filtration, adsorption, catalysis, and thermal or acoustic insulation^9,13–15^. In contrast, closed or isolated pores contribute to reduced density, enhanced thermal resistance, and improved structural stability. The incorporation of construction and demolition waste, particularly recycled concrete, as a raw material for porous ceramics offers multiple benefits. It not only provides an effective pathway for managing large volumes of waste but also contributes to sustainable resource utilization by reducing reliance on virgin raw materials. Furthermore, the transformation of concrete waste into porous ceramics aligns with the principles of circular economy and environmental protection, while simultaneously producing materials with versatile properties suitable for a wide range of engineering, environmental, and industrial applications^9–16^.

To meet industrial demand, various techniques have been developed to fabricate porous ceramics with adapted microstructures, including (i) burn-out of fugitive pore formers, (ii) direct foaming of liquid slurry, and (iii) replication of sacrificial foam templates^6^. These methods begin with a stable slurry but have limitations, such as the use of toxic polymers in water-based gel casting^9,12,13^, ice expansion or solvent-induced shrinkage in freeze forming^9,11^, and low wet-body strength in direct coagulation casting. A novel direct-consolidation method using starch as a binder has been introduced to overcome these issues^16–18^. Heating a starch-containing slurry to 55–80 °C weakens intermolecular forces, causing granules to swell significantly through water absorption. The selected binder is inexpensive, environmentally friendly, and burns out easily, though controlling pore size, geometry, and concentration remains challenging, which can be improved by adding chemical modifiers^19^. The in-situ pore-forming process offers another eco-friendly alternative, producing uniformly distributed pores through the decomposition of starting materials without generating carbon dioxide^13,15,20^. The production of bricks with improved thermal insulation and thermal shock resistance remains a significant challenge for the construction and refractory industries^21^. To overcome this limitation, numerous previous studies^21–28^ have investigated the partial or complete substitution of costly polymeric pore-forming agents, such as polystyrene, with vegetable wastes, particularly sawdust and straw. Aramide^22^ reported that sawdust additions in insulating firebricks should not exceed 15% to avoid mechanical degradation, whereas Viruthagiri et al.^23^ proposed a limit of 20%, achieving thermal conductivity values between 0.0417 and 0.1429 W m^− 1^ °C^− 1^ at 1100 °C. Cultrone et al.^24^ obtained a porosity of 60% using 10% sawdust in silica- and phyllosilicate-rich clay, although insulation efficiency was assessed qualitatively by infrared imaging. Bories et al.^25^ incorporated wheat straw, sunflower seed cake, and olive stone flour into clay bodies, achieving a cold bending strength of 10 MPa at 920 °C and a minimum thermal conductivity of 0.2 W m^− 1^ K^− 1^. Hadi and Hussein^13^ reported a minimum thermal conductivity of 0.31 W m^− 1^ K^− 1^ at 1300 °C when 30% wheat straw was used in refractory formulations. Hossein and Roy^26^ compared several pore-forming agents, including fly ash, rice husk, and rice straw ash, achieving a maximum porosity of 57% at 800 °C and a thermal conductivity of 0.65 W m^− 1^ °C^− 1^. More recently, Zharmenov et al.^27^ used rice husk as a renewable silica source to produce lightweight refractories with porosities exceeding 75% and bending strength above 1.6 MPa after firing at 1200 °C. Isinkaye et al.^28^ reported bulk densities of 0.95–1.6 g cm^− 3^ for rice husk-based bricks fired at 1200 °C; however, their reported crushing strength of 23.5 kN mm^− 2^ appears physically unrealistic.

This study aims to establish an effective and sustainable recycling route for concrete waste by repurposing it as a primary raw material in the production of porous ceramic bricks for construction applications. This research focuses on converting an abundant and environmentally problematic waste stream into functional building materials with enhanced thermal and mechanical performance. A central objective of the work is to comparatively investigate the influence of different categories of pore-forming agents on the properties of recycled concrete–based porous ceramics. These include chemical foaming agents such as baking powder and flour, biological agents such as yeast, and agricultural waste materials, including sawdust and wheat straw. The novel item of this study resides in the integrated evaluation of chemical, biological, and agro-waste foaming agents within a single recycled concrete ceramic system, enabling a systematic understanding of their roles in pore formation, microstructural development, and property optimization. The resulting materials were subjected to comprehensive physical, mechanical, and thermal characterizations, alongside phase and microstructural analyses using X-ray diffraction (XRD) and scanning electron microscopy (SEM). Through this approach, the study aims to propose a practical, low-cost, and environmentally responsible strategy for mitigating construction waste accumulation while advancing the development of sustainable porous ceramic bricks for the building industry.

## Materials and experimental method

### Starting materials

The primary raw materials used in this study included concrete waste, sand, starch, and various foaming agents. Initially, concrete waste obtained from demolished buildings was oven-dried at 110 °C for 4 h to eliminate residual moisture. The dried material was subsequently crushed using a pulverizer and sieved through a 100-mesh screen to obtain particles of uniform size. Natural sand sourced from Sinai was supplied and milled for 10 h at a rotational speed of 300 rpm. The chemical compositions of both the concrete waste and the sand are presented in Table 1 from the XRF analysis. High concentrations of calcium (CaO) and silica SiO_2_ were detected, highlighting the distinctive composition of this waste^29,30^. In contrast, the primary component of Egyptian sand is SiO_2_, which constitutes over 93% of the material.

Commercially available native corn starch and foaming agents, including food flour, yeast, and baking powder, were procured from local supermarkets. Sawdust, used as an organic pore-forming agent, was collected as wood flakes from nearby furniture workshops, while wheat straw fibers were obtained from agricultural sources in the Monophia region of Egypt. Both sawdust and wheat straw were sieved through a 40-mesh screen to ensure particle size uniformity. In addition, ammonium citrate tribasic (ACT; Alfa Aesar, USA) was employed as a dispersant in the preparation process.

**Table 1:** Chemical composition of concrete waste and sand.

Main Constituents	Concrete (wt%)	Sand (wt.)
**SiO**_**2**_	27.91	93.27
**TiO**_**2**_	0.39	0.30
**Al**_**2**_ **O**_**3**_	3.21	2.26
**Fe**_**2**_ **O**_**3**_	4.41	1.02
**MgO**	2.01	0.34
**CaO**	40.91	0.67
**Na**_**2**_ **O**	0.27	0.39
**K**_**2**_ **O**	0.25	0.60
**P**_**2**_ **O**_**5**_	0.06	0.12
**SO**_**3**_	1.03	0.16
**Cl**	0.05	0.15
**LOI**	18.47	0.69

### Preparation method

The ceramic slurry was prepared following the formulation detailed in Table 2.

Each component was incorporated in accordance with the specified batch composition. This proportion of sand was added to the porous samples to make them more rigid during the drying and firing processes^31^. The amount of water that is introduced in the ceramic slurry is 25 wt%. Ammonium citrate tribasic (ACT) was added as a dispersant at a concentration of 0.3 wt% relative to the solid content^9^. Dispersants facilitate the separation and equal distribution of particles in the mixture by diminishing the attractive forces between them, imparting comparable charges that induce repulsion^32^. The mixed powders were gradually introduced into the aqueous solution under continuous manual stirring to ensure uniform dispersion of the dispersant within the suspension. The resulting slurry was then transferred to a milling jar containing alumina milling balls with a total mass equal to twice that of the solid loading to achieve efficient mixing. An increased mass of alumina balls generates more forceful impacts, facilitating the disintegration of particle agglomerates^33^. Ball milling was carried out for 10 h at a rotational speed of 300 rpm to achieve a homogeneous distribution of the ceramic particles.

Following the milling process, starch was incorporated into the slurry to produce a starch-loaded ceramic suspension. An amount of starch equivalent to 15 wt% of the ternary powder composition was added^9^, and then the slurry was mixed for 30 min at room temperature. The starch served as a growth medium for gas bubble generation during foaming after the subject was later heated at 80 °C. The majority of starches are a combination of two polysaccharide types: a linear form known as amylose and a highly branched form called amylopectin. Amylose imparts gelling properties to starch in aqueous solutions. The glucose units comprising the polymeric chains in starch include several hydroxyl groups, hence imparting a pronounced hydrophilic nature to starch granules. These materials possess favourable characteristics like thickening, stabilization, membrane formation, and gelling. Hence, the starch acts like an adhesive viscous gel that holds the particles of ceramics together, thus improving the green strength^34^. During the process of hydration, starch absorbs water, and consequently, the volume increases. The decomposition process results in the formation of products such as hydrogen and carbon. The hydrogen and carbon react with oxygen from the atmosphere and hence form gases such as carbon dioxide and water vapor. The gases leave the ceramic body and hence leave pores in the body^35,36^.

To evaluate the influence of different pore-forming agents on the final porous structure, additional foaming materials, namely flour, yeast, baking powder, wheat straw, or sawdust, were introduced by 30 wt% (based on starch) into the slurry. Flour is mainly made up of organic materials (carbohydrates) that burn or produce gases during firing. The resulting empty spaces from these components create pores in the resulting ceramic material^37^. Yeast facilitates the fermentation of starch that has been converted into sugars. In the course of fermentation, yeast produces a gas known as carbon dioxide (CO_2_). The CO_2_ gas bubbles create pores in the ceramic material^38^. Sodium bicarbonate in baking powder produces gas when heated; it produces a gas known as carbon dioxide (CO_2_)^39^. Sawdust and straw are organic materials that burn or produce gas during firing; however, these particles are larger in size, which creates larger pores in the material^40,41^.

Each pore-forming agent was added at 30 wt% relative to the starch content. Before incorporation, the required quantity of each pore-forming agent was completely dissolved in distilled water using a 1:1 solution ratio^9^. The resulting solutions were manually mixed into the starch-loaded ceramic slurry for approximately 3 min to ensure homogeneity.

The prepared ceramic slurry for all samples was subsequently poured into molds to ensure reproducibility of the samples. The filled molds were subjected to isothermal heating at 40 °C for 1 h in an oven while covered with a glass lid. This temperature was selected as the optimal condition for fermentation and gas generation by yeast or baking powder, promoting effective foaming of the ceramic slurry. The temperature was then increased to 80 °C^9^ and maintained for 2 h to induce solidification of the liquid foam. To minimize water evaporation and prevent gas bubble diffusion, the molds were covered before and throughout the solidification stage. After consolidation, the ceramic foams were allowed to cool naturally inside the oven for 12 h prior to demolding. Drying must be carried out with care to avoid cracking and deformations, which may occur as a result of capillary effects and pressurization of water vapor. The phenomenon of drying shrinkage may be explained in terms of capillary effects present in the drying process. When the liquid starts to dry, its interface with the vapor becomes more energetic than its interface with the liquid. In order to reduce surface energy, the liquid tends to penetrate into the solid, trying to cover the interface, thus resulting in meniscus formation, which gives rise to capillary effects. The maximum value of capillary force is reached when its radius is able to fit into a pore, resulting in a compressive stress within the solid matrix, causing shrinkage^42^.

The demolded samples were initially fired at 900 °C with a heating rate of 1 °C/min for initial firing with a slow heating rate. The intermediate heating to 900 °C was employed to ensure complete decomposition of precursors and removal of volatile components, promoting phase stability, compositional homogeneity, and preventing undesired secondary phases^43^. Direct firing to the final temperature was avoided, as rapid heating could cause incomplete decomposition, uneven diffusion, and abrupt gas release, compromising phase purity and microstructure. Additionally, due to the high starch and foaming agent content, a slow heating rate is necessary to prevent internal stress and sample cracking, allowing controlled burnout of organics and preserving structural integrity.

The previous step was followed by further heating to final temperatures of 1200 °C, 1250 °C, and 1300 °C at a rate of 600 °C/h. The need for firing at a high temperature for sintering purposes, as well as for densification of porous ceramic samples, is recognized, as these factors will play a role in influencing the mechanical properties of the samples. The sintering cycle comprised a heating rate of 10 °C/min, a dwell time of 2 h at the designated temperature, and a cooling rate of 10 °C/min. The sinterability of demoulded samples may be understood in terms of the sintering mechanism, in which solid-state phase evolution occurs in three stages: initial neck formation, pore shrinkage, and finally, grain growth^44,45^. The main driving force in the solid-state sintering mechanism is the difference in chemical potential, which results from curved atomic interfaces, causing a flow of matter toward the “neck” where two particles are in contact, leading to bonding between two particles or grains as a result of matter being transported to the junction or “neck” formed between two particles. In the sintering mechanism, a neck or a narrow bridge is formed first between two particles, a phenomenon known as neck growth. With time, the bridges between the particles increase, and the bonds become stronger. The pores or voids present between the particles are eliminated due to the annihilation of the pores, which happens through various forms of diffusion, i.e., atom transfer. During the compaction process, which happens through the mechanism of diffusion, there are two main forms of the transportation of materials: (i) the transportation of the material from the surface of the particles to the neck, which results in the formation of bonds without any increase in density, and (ii) the transportation of the material from the grain boundary to the neck, which results in the formation of bonds with densification. During the sintering of solids, the sintering process involves the evolution of the microstructure through the mechanisms of neck formation, pore shrinkage, and grain growth^44,45^. The surface curvature of the particles results in a gradient of chemical potential, which causes the atoms to move from the grain boundaries or the surface of the particles to the neck, thereby causing the formation of bridges between the particles. The pores are eliminated due to the transfer of atoms, which causes densification. The transfer of materials happens either from the surface of the particles (bonding only) or from the grain boundaries (bonding with densification). According to M. N. Rahaman^46^, the sintering process happens due to the presence of the following four kinds of diffusions: bulk, surface, grain-boundary, and vapor diffusions. After the densification, the grain-boundary migration results in normal or abnormal grain growth due to the reduction of the grain-boundary energy.

**Fig. 1:**
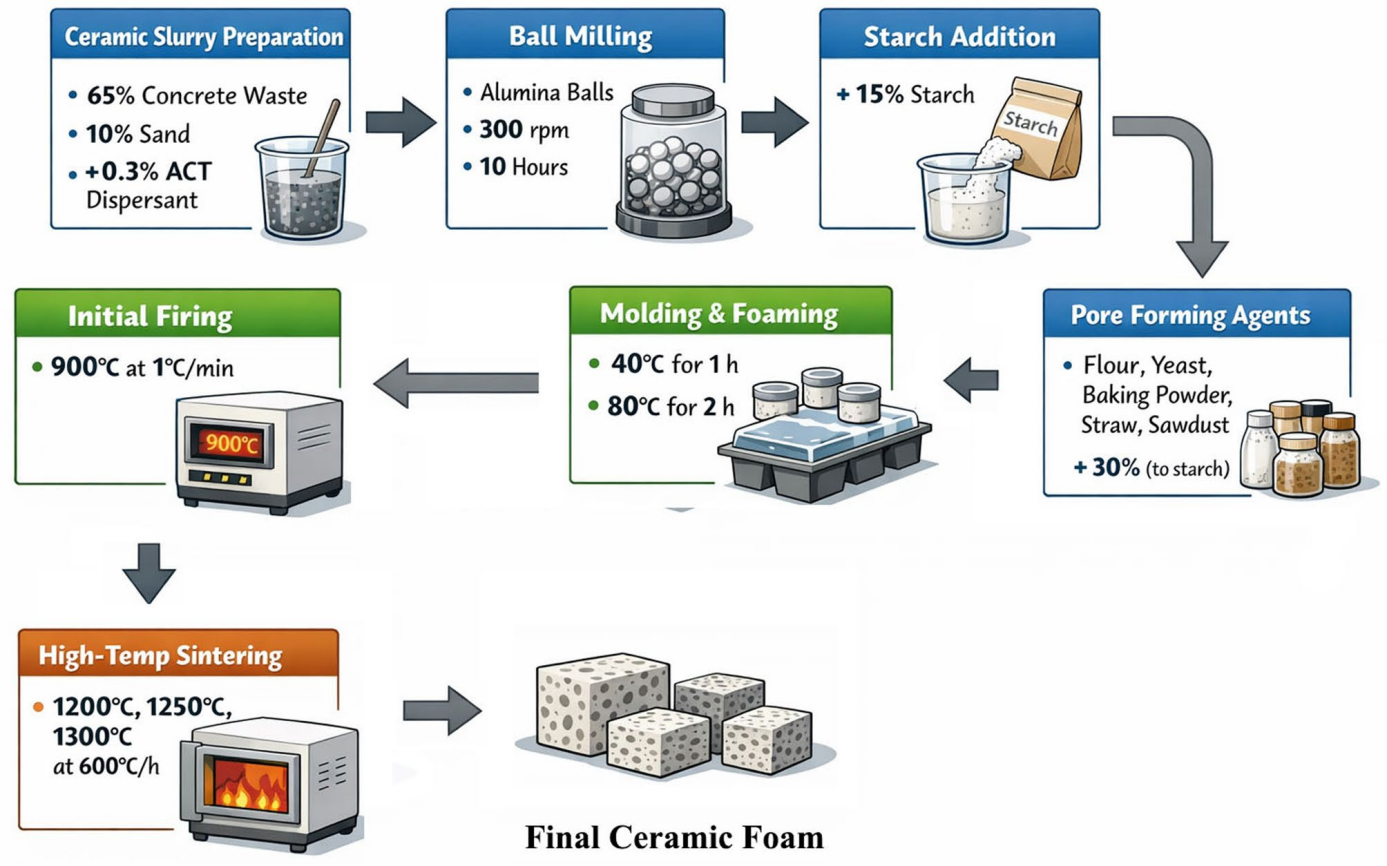
Details the steps of the preparation process.

The detailed steps of the preparation process are illustrated in Fig. 1, and the composition is listed in Table 2.

**Table 2:** The batch composition of the prepared porous ceramics.

Material	Concrete waste	Sand	water	ACT (based on concrete and sand powders)	Starch (based on concrete and sand powders)	Foaming Agent (based on starch)
**Content wt%**	65	10	25	0.5	15	30

### Characterizations

XRF uses (Axios WD XRF sequential spectrometer PANalyt-150 ical, Netherlands 2005) to ascertain the chemical composition of the waste and sand. The dried particles were thermally analysed using a Setaram Labsys TM TG-DSC16 equipment for thermogravimetric analysis (TGA) and differential scanning calorimetry (DSC). Each powdered sample was heated to 1000 °C at a rate of 10 °C per minute, and the generated thermograms showed the relevant weight changes and thermal behaviours. Using the X-ray diffraction (XRD) technique with monochromatic Cu-Kα X-ray light (λ = 1.54 Å), a model (D 500, Siemens, Mannheim, Germany) was used to determine the phase compositions at various firing temperatures. Using a step size of 0.02° and a dwell period of 1 s each step, scans were carried out throughout a 2θ range of 5° to 70°. Phase identification was performed using the International Center for Diffraction Data (ICDD, Newtown Square, PA, USA) database and a search-match method. To determine the morphology of the fired samples, a scanning electron microscopy (SEM) study was conducted using FEI, QUANTA FEG, 250. Before imaging, the samples were polished and thermally etched at a temperature 50 °C below their respective sintering temperatures for 30 min to expose grain boundaries. Then, samples were gold-sputtered using an Edwards S150A coater (England) with 1.2 kV voltage and 50 mA current in a 0.1 torr vacuum to maximize imaging. The Archimedes water displacement method determined the apparent porosity and bulk density per the American Society for Testing and Materials (ASTM C373-88) 61 standards^47^. In this test, the saturated samples were first weighed in air (Ws) and then again when immersed (Wi) before being submerged in water. The samples were dried overnight at 110 °C, and their dry weight (Wd) was noted. The following formulas were used to determine the samples’ apparent porosity (AP) and bulk density (BD) using the specific gravity of water (γ)^47^:

1 $$\:Ap=\frac{Ws-Wd}{Ws-Wi}\:x\:100\:$$

2 $$\:BD=\frac{Wd}{Ws-Wi}\:x\:\gamma\:\:$$

The compressive strength (CCS)^48^. was evaluated using the Tinius Olsen Universal multi-testing machine (UK) model 25ST running at a crosshead speed of 0.5 mm/min. The ASTM C 1424–19 standard was used to evaluate the cold crushing strength (CCS) of samples measuring 1.23 cm^2^ in area and 1.5 cm in length. The thermal transmittance (U) was determined using on-site measurements following the heat flow meter method. This approach assumes that the U-value can be calculated by dividing the average heat flow rate density by the average temperature difference, with the average taken over an extended period. The U-value estimate is given by the formula $\:\mathrm{U}=\frac{\mathrm{q}}{(\mathrm{T}\mathrm{i}-\mathrm{T}\mathrm{e})}$^49^, , where q represents the density of heat flow rate, T_i_ is the interior surface temperature, and T_e_ is the exterior surface temperature.

The Fourier Transform Infrared (FTIR) analysis of the synthesized samples was conducted at room temperature using a computerized spectrometer (model FTIR–4600, JASCO Corp., Japan) over the 4000 –400 cm^-1^ spectral range. The conventional potassium bromide (KBr) disc method was employed for the measurements. In this procedure, the prepared samples were first finely ground into a powdered form, after which approximately 2 mg of the sample powder was thoroughly mixed with 200 mg of spectroscopic-grade KBr to ensure uniform distribution. The resulting mixture was then compressed under an applied pressure of 5 tons/cm^2^ using a suitable die, forming transparent and homogeneous pellets suitable for the measurement. These pellets were subsequently used directly for FTIR measurements, allowing the vibrational characteristics of the material to be analysed.

## Results and discussion

### Thermal analysis

**Fig. 2:**
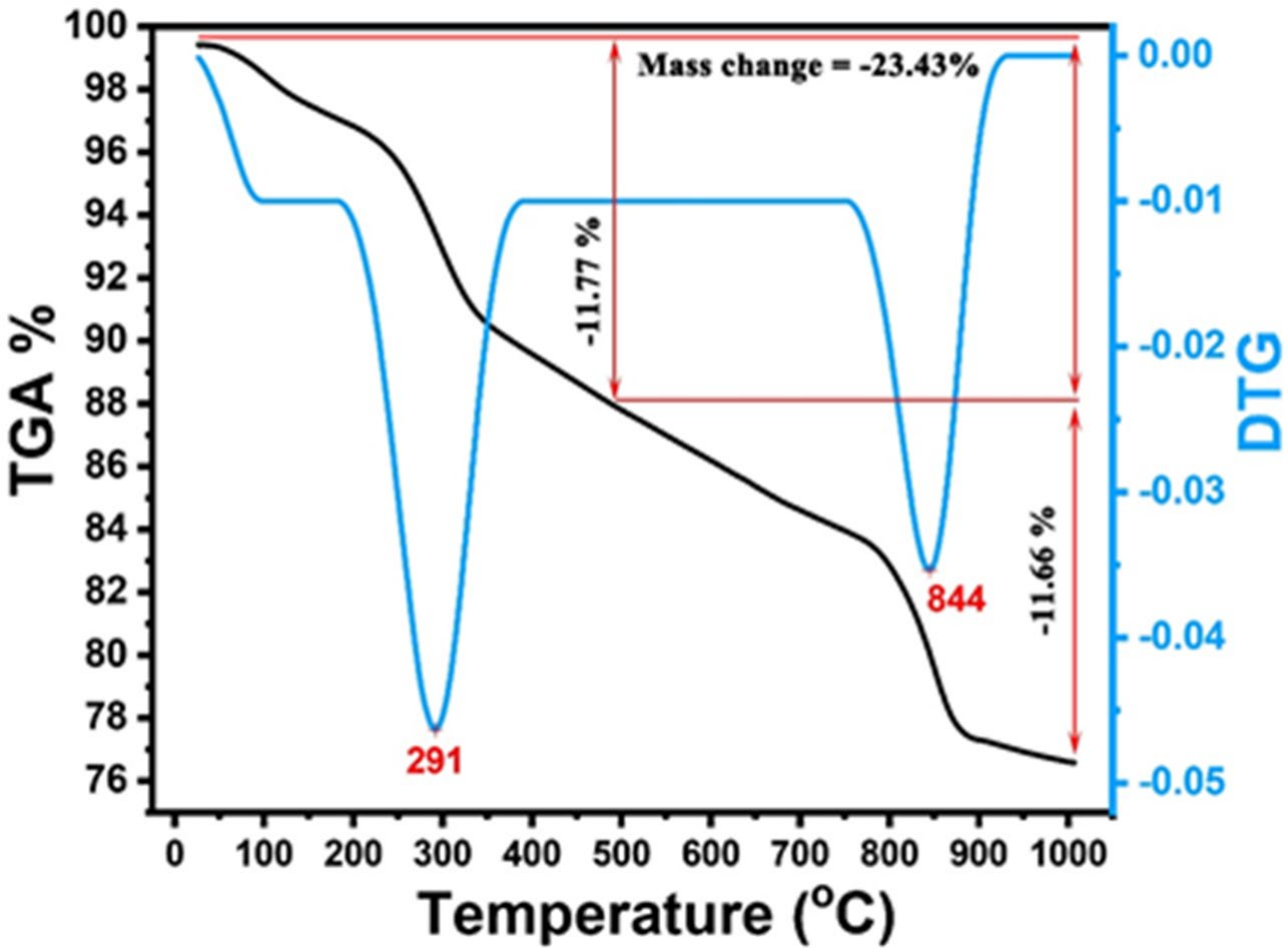
Thermogravimetric analysis and differential thermal analysis of the green body.

The selected sintering system plays a crucial role in determining the sintering behaviour of porous ceramics. As shown in Fig. 2, thermogravimetric analysis and differential thermal analysis of the green body are used to estimate the sintering system of porous ceramics.

The TGA plot shows approximately 23.43% mass loss up to 1000 °C, confirming thermal stability for sintering/foaming. The decomposition occurs in two main stages: the first major weight loss takes place between about 200 and 320 °C, with a peak around 291 °C, where the mass drops from approximately 94–95% to around 78%, corresponding to a loss of about 16–17%. The second stage occurs between roughly 750 and 900 °C, with a peak near 844 °C, during which the mass decreases from approximately 84–85% to about 76–77%, representing an additional loss of approximately 7–8%. Overall, the TGA profile indicates a total mass loss of about 23.43– 24% by 1000 °C.

Regarding the previous data analysis, the elimination of absorbed water^]^ and the dehydration processes of various hydrates (C-S-H, carboaluminates, ettringite, etc.)^51^ resulted in an endothermic peak with a mass change of about 5% weight loss at temperatures between 100 °C and 200 °C, according to the differential thermal curve. The DTA curve displayed an endothermic peak at around 280 °C, when the starch broke into fragments^36^. Furthermore, at 450–500 °C, a mass shift of 11% weight loss is noted, which is associated with the dehydroxylation of portlandite, another hydration product, and the breakdown of starch into CO_2_ and steam, along with minor endothermic peaks^13^. The peak of the C-S-H reaction’s dehydration diminishes as the prior heat treatment increases and vanishes for heat treatments above 300 °C. These findings concur with those found in Refs.^52,53^. At about 600 °C, the differential thermal curve displayed an upward exothermic peak, indicating the reaction of carbon and oxygen in the breakdown of organic matter. In contrast, the thermogravimetric curve tended to be horizontal^36^. The decarbonation of calcium carbonate from the clinker and filler is responsible for the 23% mass change in weight loss, accompanied by an endothermic peak at 850 °C. As expected, the decarbonation of calcium carbonate totally vanishes for heat treatments above 850 °C^52^. The temperature history of concrete exposed to a fire regime can thus be ascertained using this reaction as a marker. Even after an 800 °C heat treatment, the second peak, which represents the dehydroxylation of portlandite, does not entirely vanish. During furnace cooling after heat treatment, the amorphous portion of portlandite recrystallized^54^.

### Physical properties

**Fig. 3:**
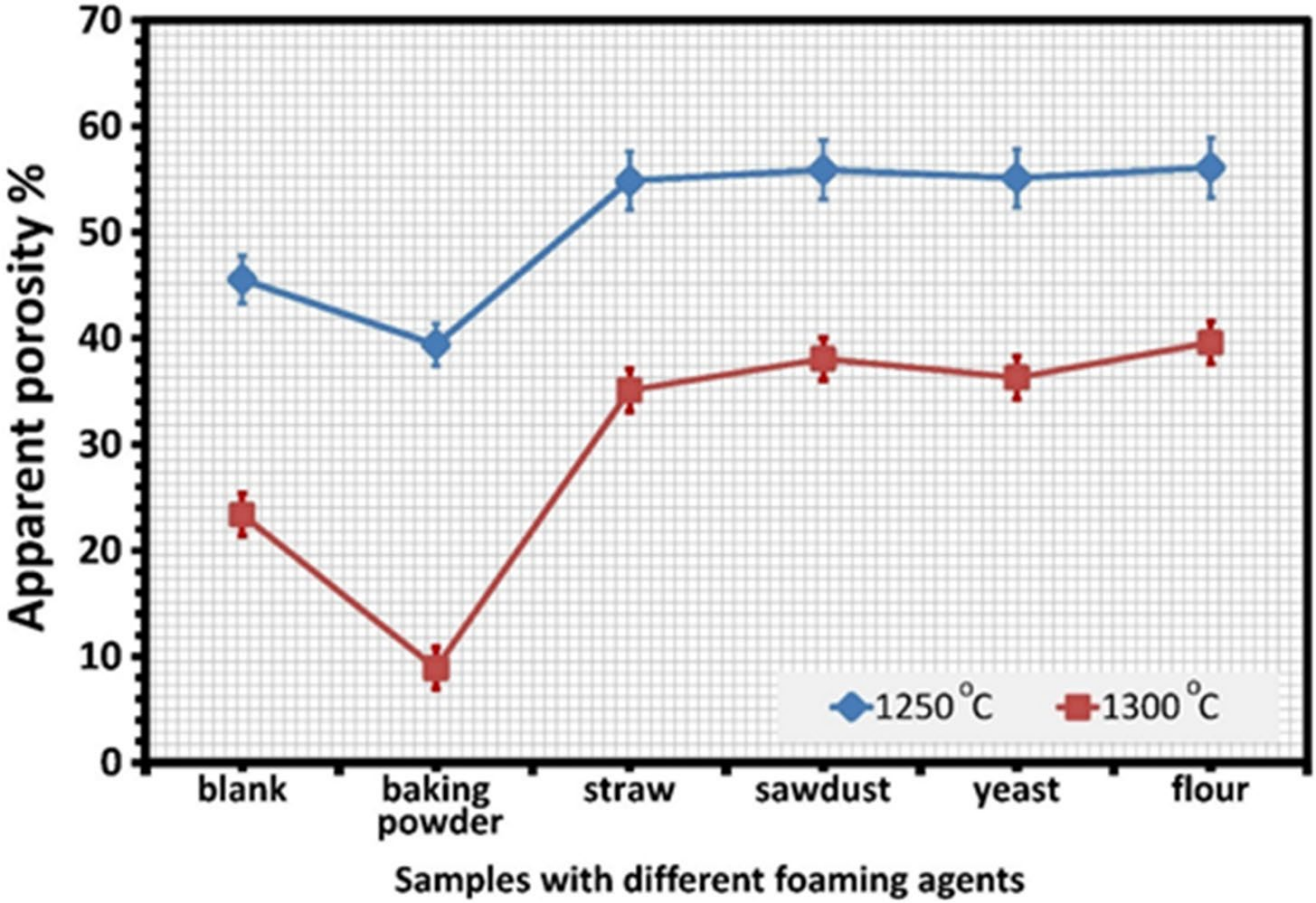
The apparent porosity of samples with different foaming agents at 1250 °C.

**Fig. 4:**
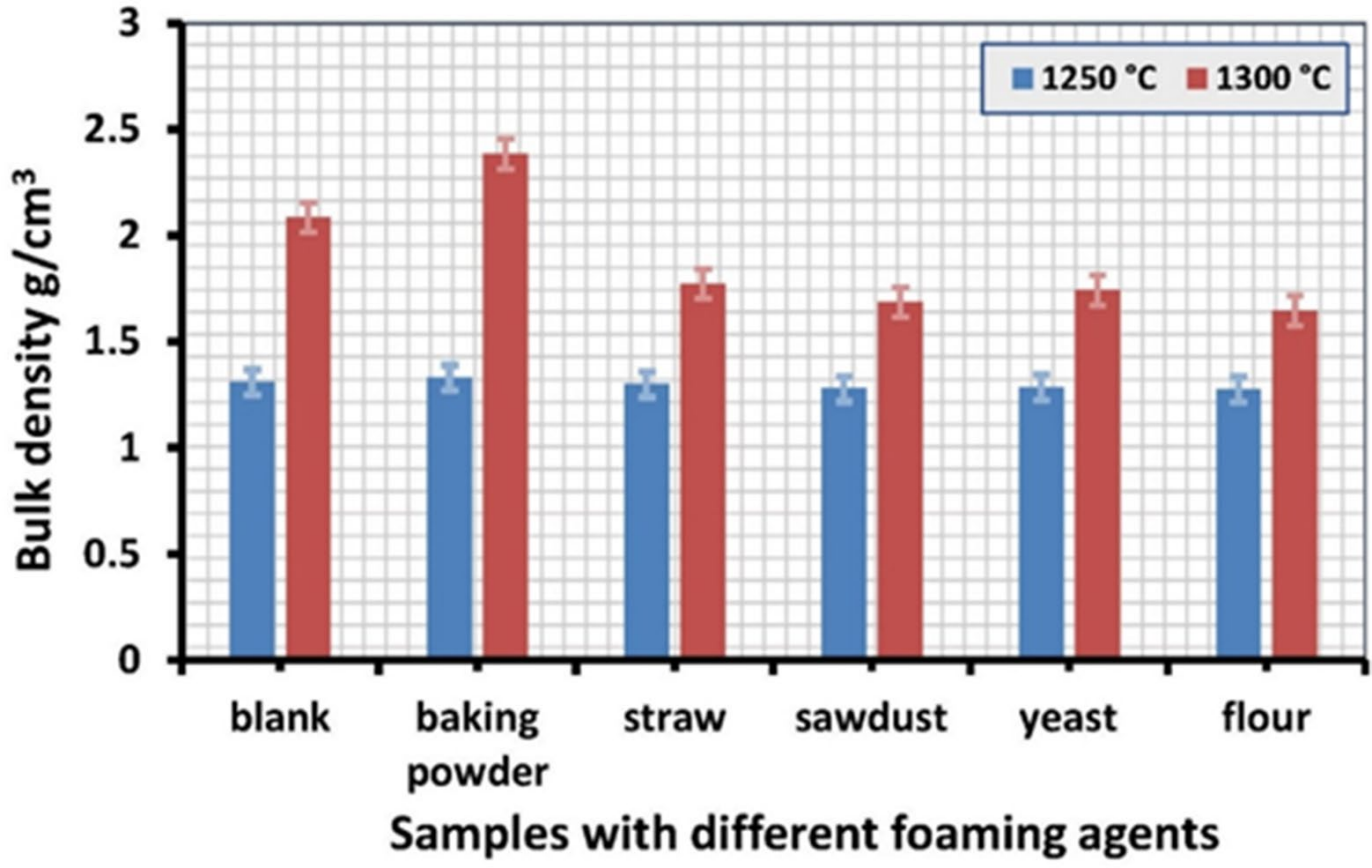
The bulk density of samples with different foaming agents at 1250 °C and 1300 °C.

Figures 3 and 4 show the physical characteristics of bulk density and apparent porosity, respectively. After firing at 1200 °C, all samples are Fragile and subject to cracking because they are not fully sintered. As the firing temperatures rise, the sintering ability grows from its initial appearance at 1250 °C.

When flour or yeast was added as pore-forming agents with starch, the density decreased to a minimum value. It ranged from 1.27 g/cm^3^ to 1.28 g/cm^3^ at 1250 °C and from 1.64 g/cm^3^ to 1.74 g/cm^3^ at 1300 °C, respectively. The apparent porosity of these samples was between 56% and 55% at 1250 °C and 39% to 36% at 1300 °C, respectively. Compared to samples containing starch or yeast, flour samples exhibited higher porosity.

and 1300 °C.

Thus, unlike pure starch, flour especially wheat flour can contribute to porosity through gas release during firing and protein-assisted foaming. As a result, the pore produced by flour is primarily caused by protein-assisted foaming, which results in foam bubbles, rather than just the swelling (and gelatinizing) of starch granules. On the other hand, when yeast is employed as a pore-forming agent, it creates gas during fermentation, which causes pores to form inside the ceramic structure^55^. This is because more yeast reacts with the starch to create carbon dioxide bubbles, which are a foaming agent. In the presence of some starch, the porous ceramics made by biological foaming showed noticeably greater open porosities, as seen in Fig. 3. Pore networks are created when the bubbles formed during foaming consolidate or connect. Conversely, the closed porosities are tiny and mostly unaffected by the amount of starch. This suggests the development of highly three-dimensional interconnected pore spaces^9,56,57^ made up of big pore bodies joined by tiny hole throats.

However, samples containing straw (or sawdust) have less density and higher porosity; samples containing straw have 54%, 1.29 g/cm^3^ at 1250^°^C and 35%, 1.77 g/cm^3^ at 1300 °C, while samples containing sawdust have 55%, 1.27 g/cm^3^ at 1250 °C and 38%, 1.68 g/cm^3^ at 1300 °C. These samples are examples of biomass materials that can make porous ceramics. When mixed into ceramic materials, these biomass elements are burned away during firing, leaving pores that raise the end product’s porosity. Furthermore, these samples’ high porosity results from pore collapse brought on by starch, sawdust, or straw^58^. Because sawdust or straw particles have a larger variation of particle sizes, their unequal pore diameters translate into increased porosity. It is commonly known that when sawdust or straw breaks down during the firing process, significant volumes of CO and CO_2_ gases are released. As a result, these gases create pores, reducing leftover concrete particles’ density^59,60^.

When baking powder is used as a pore-forming agent in samples, it also breaks down during the sintering process, releasing water vapour and carbon dioxide. This is because baking powder is not a pure chemical product but rather a blend of substances. To absorb moisture and avoid an untimely reaction, it usually comprises sodium bicarbonate (the base, NaHCO_3_), one or more acidic salts, such as potassium bitartrate, sodium acid pyrophosphate, or monocalcium phosphate, and a filler, like cornstarch. Low temperatures (39% at 1250 °C) can produce a foaming effect and a more porous structure. Sodium or calcium silicate, which can serve as a sintering aid and promote liquid phase sintering, is formed at high temperatures (1300 °C). This can result in the smallest increase in bulk density compared to other samples, with porosity reaching 8.9% and bulk density reaching 1.33 g/cm^3^ at 1300 °C^61^. It is commonly known that sodium diffuses into the sample surface layers in industrial alumina powders. At high temperatures (about 780 °C), Na and SiO_2_ combine to generate a low-melting eutectic that lowers the viscosity of quartz glass and facilitates sintering^62^.

**Table 3:** The descriptive analysis of the porosity and density of the prepared samples at 1250 °C and 1300 °C.

Analysis	Temp.	N	Mean	SD	SEM	Median	Test statics		
							T-static	DF	Prop>ǀtǀ
**Porosity**	1250 °C	6	51.15	7.01	2.86	54.98	10.12	5	1.6 × 10^− 4^
	1300 °C		30.22	11.93	4.87	35.70			
**Density**	1250 °C		1.29	0.02	0.008	1.29	-5.34	5	0.003
	1300 °C		1.88	0.29	0.118	1.75			

The statistical analysis represented in Table 3 reveals a significant influence of sintering temperature on the porosity and density of the prepared samples. The porosity decreased markedly from 51.15 ± 7.01% for samples sintered at 1250 °C to 30.22 ± 11.93% at 1300 °C, corresponding to an average reduction of approximately 21%. The paired sample *t*-test yielded a t-value of 10.12 with 5 degrees of freedom and a p-value of 1.6 × 10^− 4^, which is far below the significance level (α = 0.05), indicating that the difference between the two temperatures is statistically significant and confirming that higher sintering temperature promotes considerable densification of the material. The relatively larger standard deviation observed at 1300 °C suggests increased variability in pore collapse, which may be associated with intensified diffusion mechanisms and viscous sintering processes that enhance particle rearrangement, neck growth, and pore coalescence at elevated temperatures. Regarding density, the samples sintered at 1250 °C exhibited an average value of 1.29 ± 0.02 g/cm^3^, while those sintered at 1300 °C showed a mean density of 1.88 ± 0.29 g/cm^3^. The small standard error of the mean indicates good measurement precision. The statistical evaluation produced a t-statistic of -5.34 with 5 degrees of freedom and a p-value of 0.003, confirming a statistically significant difference between the densities at the two temperatures. However, the negative *t*-value and the higher average density at 1300 °C are due to the observed reduction in porosity, which may indicate compositional effects, or structural changes occurring at higher temperatures. In some cases, elevated sintering temperatures can induce microstructural phenomena such as phase separation, pore bloating, or localized expansion, which may influence the apparent bulk density despite the reduction in overall porosity. Nevertheless, the strong reduction in porosity together with the statistically significant temperature effect demonstrates that 1300 °C plays a crucial role in modifying the microstructure and densification behavior of the prepared samples. The large statistical effect size associated with the porosity change further highlights the strong impact of temperature on microstructural consolidation, which is particularly important for materials intended for optical or luminescent applications, where reduced porosity contributes to improved structural compactness and minimizes scattering losses.

### Compressive strength

**Fig. 5:**
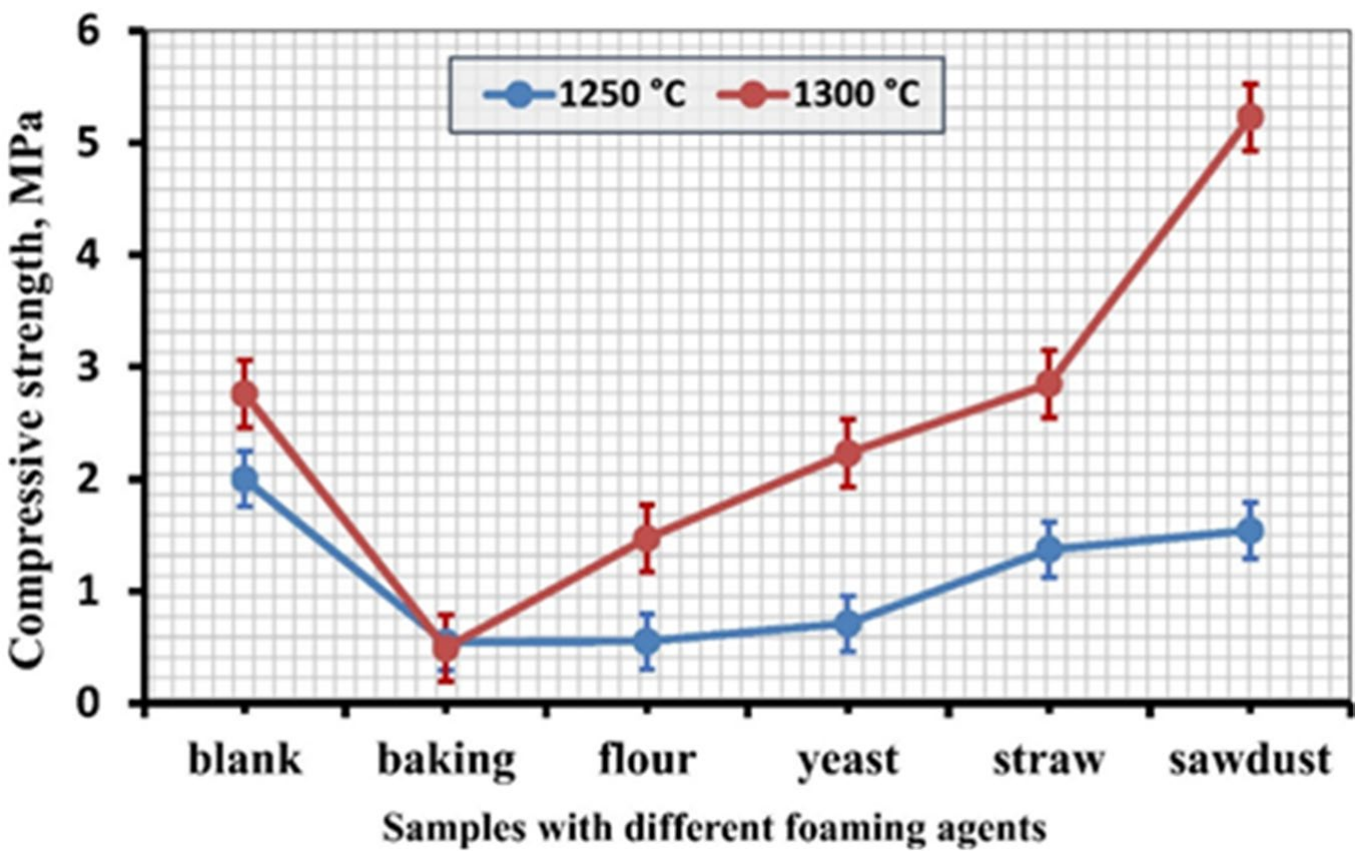
The compressive strength of samples with different foaming agents at 1250 °C and 1300 °C.

The effect of various pore agents on the compressive strength of porous ceramic is depicted in Fig. 5. Raising the firing temperatures increases the compressive strength. As a result, samples containing starch had an average compressive strength of 2 and 2.76 MPa, baking powder of 0.5 and 0.4 MPa, flour of 0.55 and 1.47 MPa, yeast of 0.71 and 2.23 MPa, straw of 1.37 and 2.85 MPa, and sawdust of 1.54 and 5.23 MPa, at 1250 °C and 1300 °C, respectively. These outcomes are consistent with research by previous studies^63,64^, which found that when porosity increases, the material’s mechanical strength decreases. Porosity is an essential characteristic that significantly affects how materials behave mechanically, especially their deformation characteristics. Materials’ structural integrity usually deteriorates with increasing porosity, changing their ability to support loads. Porous ceramics’ mechanical strength is also influenced by the size and microstructure of their pores^65–68^. A ceramic body’s mechanical strength will decrease with increasing pore size^69,70^. The pores’ geometry may result in stress concentration points, which could worsen the consequences of mechanical loading and hasten failure^71,72^. Mechanical strength decreased significantly due to transforming spherical pores into solid sphere structures or ellipsoidal shapes. This reduction is probably brought on by the greater concentration of tension at the ellipsoidal pores’ margins, which is more noticeable than in spherical ones.

As observed later in SEM, the linked pores that distribute stress and stop cracks from spreading give sawdust-containing samples their high strength^73^. However, compared to samples containing sawdust, samples containing straw exhibit the development of cracks because the composition of straw contains extra silica, which promotes the formation of cristobalite at high temperatures and decreases strength^74^. β-cristobalite, which is stable at high temperatures, and α-cristobalite, which is stable at lower temperatures, are the two types of cristobalite. A significant volume shift occurs from the high-temperature β-form to the low-temperature α-form, creating internal stresses and microcracks in the ceramic material that reduce strength^74^.

Baking powder-containing samples lose strength because of alkali metal ions, particularly Na ions^62,73,75,76^, which encourage the crystallization of cristobalite. The sintering process causes Na to diffuse into the sample surface layers, which results in a much larger cristobalite fraction in the surface layer than in the interior. The cristobalite phase crystallization in the surface layer was more successful throughout the sintering process, and the volume contraction resulted in the most severe fissures on the sample surface. Because cristobalite crystallization blocks the viscous flow of quartz glass, it can stop additional sintering of the glass. Less fused viscous low quartz glass is present in a sample with a higher percentage of crystallized cristobalite. The cristobalite phase also lessens the quartz glass’s viscous flow. The crystallization of cristobalite starts in large quantities. Over time, the sample’s expansion surpasses its contraction, and the fused quartz progressively changes into cristobalite^62^. This causes samples to lose strength, particularly around 1300 °C, as the amount of cristobalite increases, as can be shown later in the XRD section.

**Table 4:** The descriptive analysis of the compressive strength of the prepared samples at 1250 °C and 1300 °C.

Analysis	Temp.	N	Mean	SD	SEM	Median	Test statics		
							T-static	DF	Prop>ǀtǀ
**compressive strength**	1250 °C	6	1.11	0.60	0.24	1.04	-3.98	5	0.0105
	1300 °C		2.83	1.27	0.51	2.62			

The statistical analysis of the compressive strength presented in Table 4 shows that sintering temperature significantly influences the compressive strength of the prepared samples. The samples sintered at 1250 °C exhibited a mean compressive strength of 1.11 ± 0.60 with a median of 1.04, indicating relatively low mechanical resistance. In contrast, the samples sintered at 1300 °C showed a considerably higher mean compressive strength of 2.83 ± 1.27 with a median of 2.62, representing more than a twofold increase in strength. The paired *t*-test yielded a t-value of − 3.98 with 5 degrees of freedom and a p-value of 0.0105, which is below the significance level (α = 0.05), confirming that the increase in compressive strength with temperature is statistically significant. This improvement can be attributed to enhanced densification and stronger particle bonding at higher temperatures, which reduce internal pores and defects that act as stress concentrators. The slightly higher variability observed at 1300 °C may be related to localized microstructural changes such as grain growth or uneven pore elimination during sintering. Overall, the results demonstrate that increasing the sintering temperature to 1300 °C significantly enhances the mechanical strength of the samples due to improved microstructural consolidation.

### SEM analysis

**Fig. 6:**
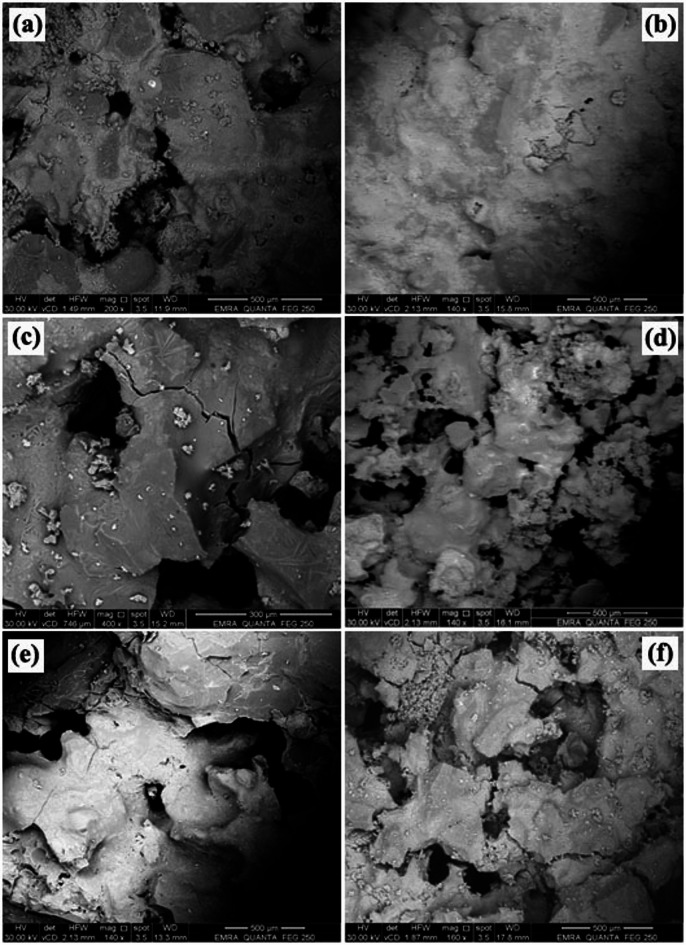
The microstructure of samples with various foaming agents firing at 1250 °C: **a**) starch, **b**) baking powder, **c**) straw, **d**) sawdust, **e**) yeast, and **f**) flour.

**Fig. 7:**
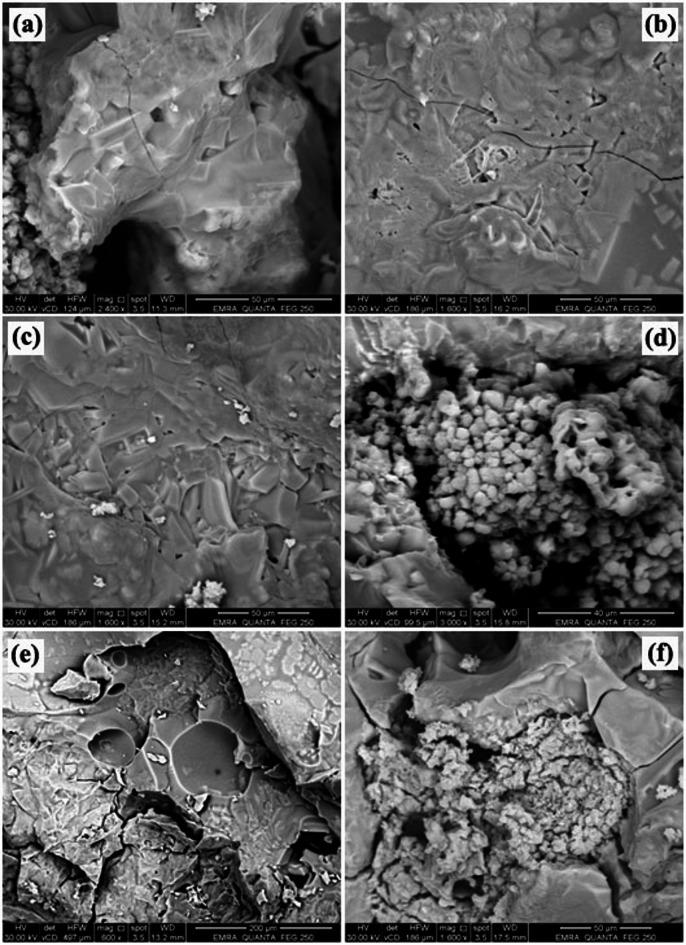
The microstructure of samples with various foaming agents firing at 1300 °C: **a**) starch, **b**) baking powder, **c**) straw, **d**) sawdust, **e**) yeast, and **f**) flour.

Figures 6 and 7 depict the microstructure of the prepared samples following firing at 1250 °C and 1300 °C. Concerning the effect of firing temperature, the microstructural examination revealed a significant impact on pore evolution and interconnectivity. In particular, samples sintered at 1300 °C (Fig. 7) developed a closed and well-interconnected structure. Relative to the specimen fired at 1250 °C (Fig. 6), the sample treated at 1300 °C showed a modest decrease in pore formation and a slight reduction in total porosity. This behavior can be attributed to enhanced densification at the higher firing temperature, which promotes particle rearrangement and mass transport mechanisms such as lattice and grain boundary diffusion^77^. Consequently, pore shrinkage and partial pore closure occur, leading to a more consolidated structure. In contrast, firing at 1250 °C appears to provide insufficient thermal energy for complete densification, resulting in relatively higher pore volume and a less compact microstructure^78,79^.

Conversely, the experimental observations clearly demonstrate that the type of foaming agent exerts a significant influence on the resulting pore structure of the prepared samples. Specifically, specimens formulated with baking powder exhibited the smallest average pore size in comparison to those produced using alternative foaming agents. The other agents investigated, namely flour, sawdust, straw, and yeast, generated comparatively larger pore structures under similar preparation conditions. This variation in pore morphology can be attributed to differences in the decomposition mechanisms, gas release rates, and thermal stability characteristics associated with each foaming agent^80^. Therefore, the selection of baking powder as a foaming agent appears to promote the formation of a finer and more uniformly distributed pore network relative to the other evaluated materials.

Microscopic analysis of the porous microstructure revealed that the majority of the porosity consisted of intact cell struts and deformed pore structures with a wide range of pore sizes^9^. These pores were predominantly interconnected, forming a continuous network throughout the ceramic matrix. It is well established that the mechanical strength of porous ceramics can be significantly enhanced by the presence of interconnected porosity. Unlike closed pores, interconnected pores facilitate more uniform stress distribution within the structure and can effectively hinder crack initiation and propagation, thereby improving overall structural integrity^81,82^. Accordingly, the sample exhibiting the highest total porosity specifically the specimens containing wheat straw or yeast demonstrates a microstructure characterized by a highly interconnected pore network, which contributes to its enhanced mechanical performance.

On the other hand side, the pore size, pore dispersion, and total porosity of these samples are affected by the swelling behaviour of these chemicals in addition to starch during processing. Large spherical cavities produced by these pore agents are visible in these samples. These agents may swell during processing, resulting in voids that can be eliminated by heat treatment later on, leaving behind pores^83^. Open and closed pores are the two types of pores seen in Fig. 7e and f. The kind and quantity of pore former, as well as the processing conditions, affect this observation^84^.

### XRD analysis

**Fig. 8:**
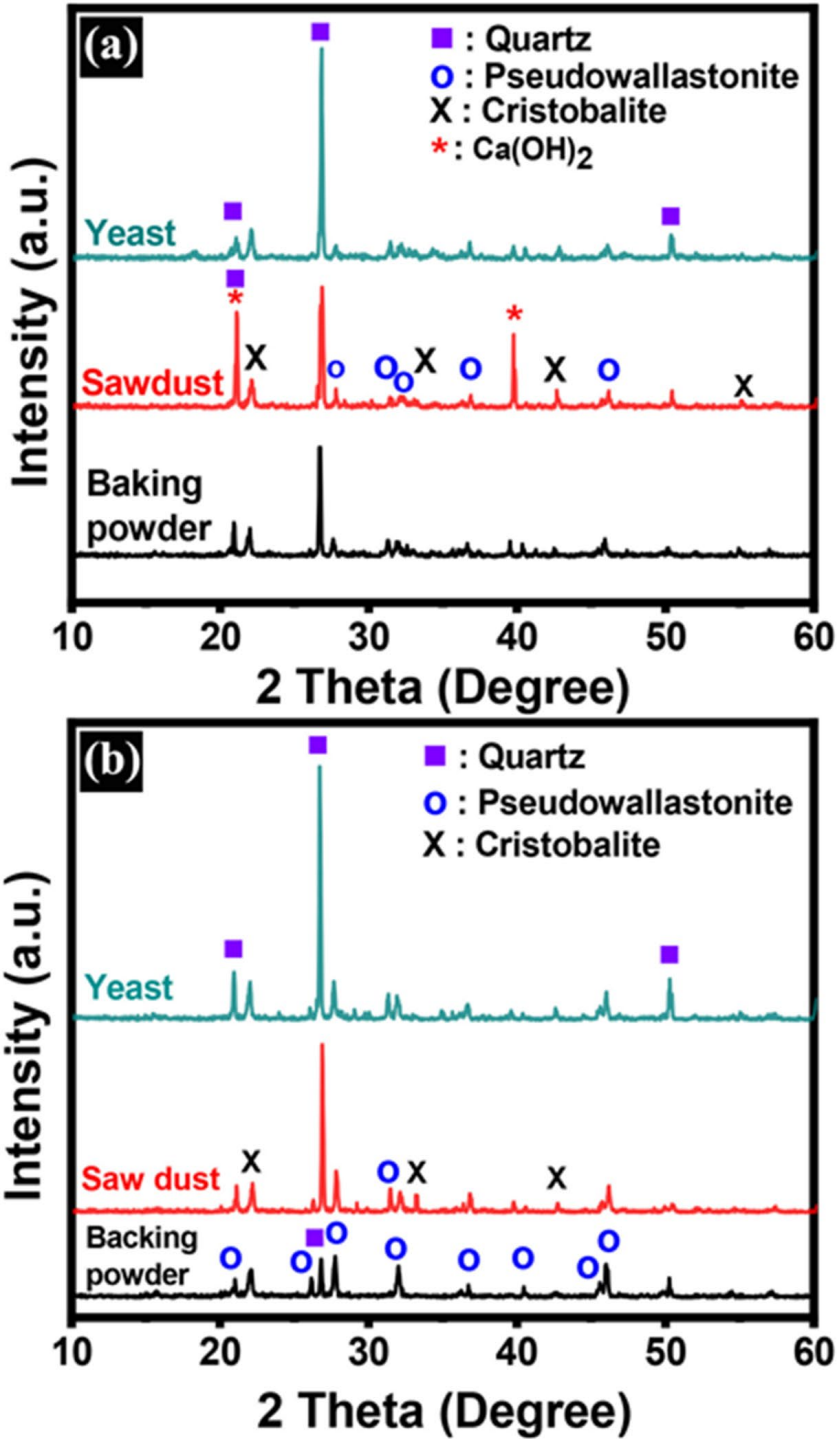
XRD pattern of baking powder, sawdust, and yeast samples fired at **a**) 1250 °C and **b**)1300 °C.

Figure 8 displays the XRD pattern of a few chosen samples that were fired at 1250 °C and 1300 °C. When the temperature reached 1250 °C, samples that contained baking powder, yeast, and sawdust showed a small amount of α-CaSiO_3_ (PDF#89-6463), along with the appearance of quartz (PDF#87–0703) and some cristobalite (PDF#82-1406). The formation of cristobalite can be attributed to the addition of sawdust that significantly altered the microstructure and firing behavior of the body.

Pereira et al.^85^ assumed that during heating, the foaming agent like saw dust burns out between approximately 200 and 600 °C, leaving behind a highly porous structure that increases internal surface area and enhances oxygen diffusion and heat transfer throughout the sample. This increased porosity reduces mechanical constraints within the matrix, allowing silica particles greater structural freedom to reorganize at high temperatures. The combustion of sawdust can also generate localized exothermic reactions, creating micro-hotspots that may temporarily exceed the bulk furnace temperature, thereby promoting the transformation of quartz (SiO_2_) into cristobalite, which typically forms above 1100–1200 °C. Additionally, the porous network and associated microstructural defects act as nucleation sites, lowering the energy barrier for cristobalite crystallization^85^.

Calcite formation and its subsequent transformation into calcium hydroxide were observed only in the sawdust-containing ceramic sample (Fig. 8a) because the incorporation of sawdust altered both the chemical reactions and microstructural development during firing up to 1250 °C^85–87^. The combustion of sawdust releases significant amounts of CO_2_ and produces a highly porous structure, enhancing gas diffusion and creating localized reactive environments within the ceramic matrix. Many previous studies^24,88,89^ postulated that, in the presence of calcium-bearing phases such as CaO, the evolved CO_2_ can promote carbonation reactions, leading to the formation of calcite (CaCO_3_) at intermediate temperatures. The increased porosity facilitates effective interaction between CO_2_ and calcium species, intensifying this process. At higher temperatures approaching 1250 °C, calcite decomposes to CaO, which, upon cooling and exposure to moisture, readily hydrates to form calcium hydroxide (Ca(OH)_2_). Conversely, denser samples without sawdust restrict gas diffusion and limit carbonation and subsequent hydration reactions. Thus, CO_2_ generation, enhanced porosity, and increased calcium phase reactivity collectively account for the exclusive formation of calcite and its transformation to calcium hydroxide in the sawdust-modified ceramics.

The peak intensity of CaSiO_3_ for the sample containing baking powder increases noticeably when the temperature rises above 1300 °C. This implies that the preferred orientation and crystal structure of CaSiO_3_ may have changed throughout the heating process. The peak intensity of CaSiO_3_ at these two locations increases noticeably when the temperature rises above 900 °C. Between 800 °C and 900 °C, there may be a significant conversion of calcium silicate hydrate C-S-H to CaSiO_3_, which could explain this transition^90^. The fact that baking powder contains more α-CaSiO_3_ than other samples may be because sodium acts as a flux, which lowers the sintering temperature needed to create calcium silicate (CS)^91^. In the present work, the sample containing baking powder exhibits a high peak intensity of α-CaSiO_3_ upon firing at 1300 °C. The peak intensity of α-CaSiO_3_ rises, falls, and then rises once again. The peak at about 2θ = 45.5^°^, on the other hand, keeps rising, and both peaks move somewhat in place.

### Thermal transmittance (U) and thermal conductivity

The ability of a material to transmit heat directly relates to its thermal transmittance, which is designated as the U value. The thickness of the material determines how much heat can transfer through it under steady-state conditions. The U-value represents the heat transfer rate through a unit area per unit time, divided by the temperature difference between inside and outside spaces. The U-values are determined using methods described in several earlier studies^92–94^.

**Fig. 9:**
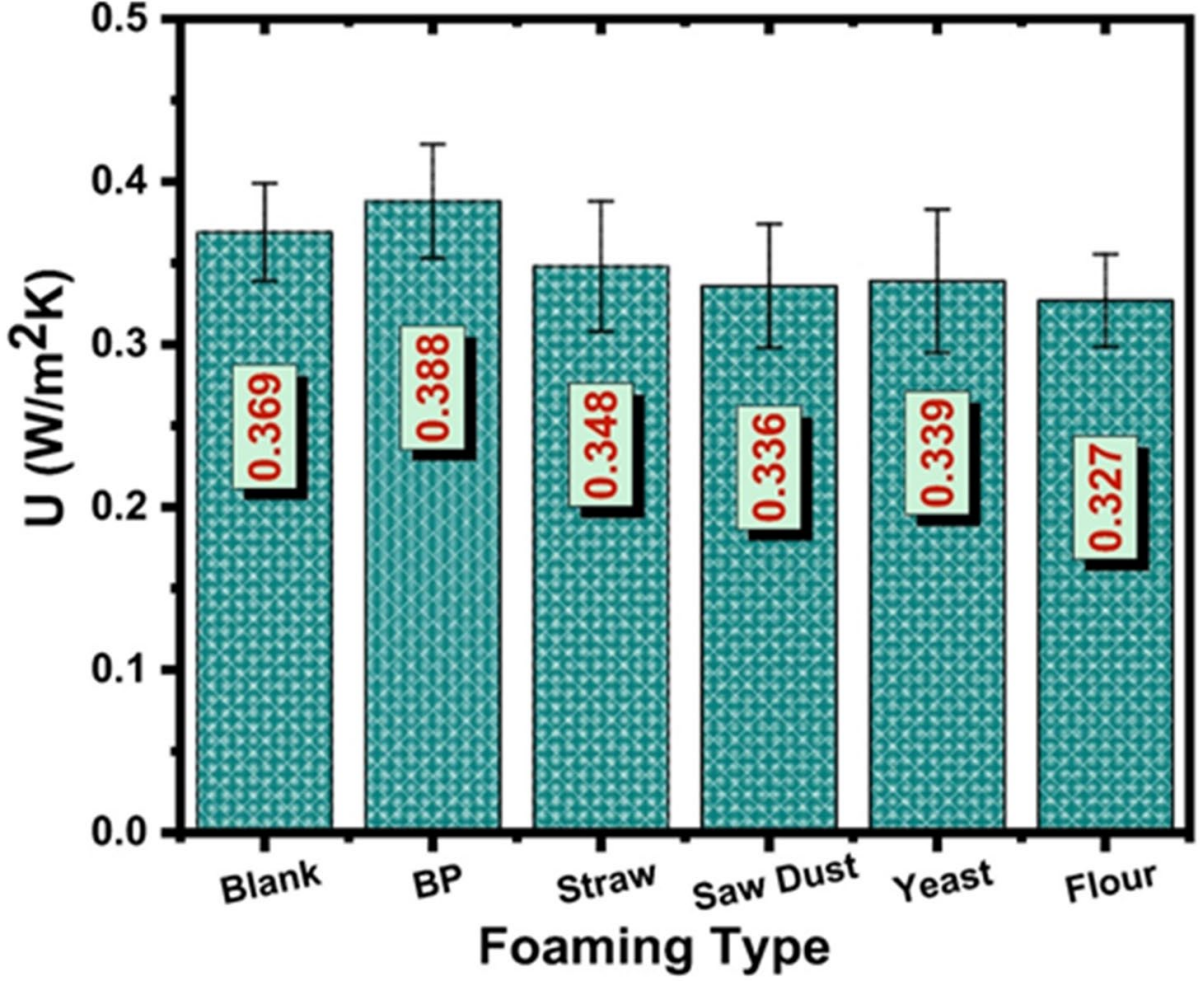
The variation of thermal transmittance with the natural foaming agent type.

The results in Fig. 9 demonstrate how different natural foaming agents influence the preparation of porous materials derived from crushed construction waste after firing at 1250 °C, based on their estimated thermal transmittance values (U-values). The U-value of the prepared samples ranged from 0.327 to 0.388 W/m²K, showing only a slight variation between samples depending on the type of foaming agent used. The U-value of the foaming agent BP (baking powder) sample was the maximum value at 0.388 W/m^2^K. The corresponding U-values of the blank, straw, yeast, sawdust, and flour were decreased slightly to 0.369, 0.348, 0.339, 0.336, and 0.327 W/m²K, respectively. On the other hand, the thermal conductivity values, estimated using the method described in reference^95^, exhibit noticeable variation among the tested samples, highlighting the effect of their composition and internal structure on heat transfer behavior. The blank sample exhibited a thermal conductivity of 0.044 W/m.K, while the sample containing baking powder showed a slightly higher value of 0.0465 W/m.K, indicating relatively greater heat transfer. In contrast, the incorporation of natural and porous additives led to reduced thermal conductivity values, with straw and sawdust recording 0.0417 and 0.0402 W/m.K, respectively. Further reductions were observed for the yeast and flour samples, which demonstrated values of 0.0406 and 0.0392 W/m.K. The obtained results suggest that the addition of organic and pore-forming materials enhances thermal insulation performance by lowering thermal conductivity, likely due to increased porosity and air entrainment within the matrix.

Regarding the thermal transmittance (U-values), the natural foaming agent plays a key role in shaping the porosity of the prepared samples, which in turn affects how well they can insulate against heat. Because the foaming agent influences how pores form and spread throughout the material, the U-value changes according to its activity. Temperature also significantly impacts the process, affecting how the foaming agent behaves during preparation and how stable the pores remain. So, how the natural foaming agent works together with temperature changes ultimately causes variations in the ability of the material to resist heat flow. Hence, flour as a foaming agent positions its performance between the chemical and fibrous natural agents.

Flour can trap gases produced during processing, forming pores with less uniformity. Previous studies^96,97^. highlighted that flour-based biofoams typically require optimization through additives or process controls to enhance pore stability and distribution. Hence, its moderate insulating capability in this work may be improved with applied temperature^92–98^.

Incorporating agricultural residues such as straw and sawdust confirms the feasibility of using lignocellulosic materials as foaming agents to introduce porosity into construction composites. The fibrous nature of straw and sawdust inherently creates larger pores and interconnected voids, increasing permeability to convective heat transfer. This matches observations from previous work by Ulutas et al.^98^, where using natural fibers improved porosity and eco-friendliness but compromised thermal performance compared to more controlled biological or chemical foaming methods. The use of such fibers is often favoured in green building contexts due to their availability and environmental benefits, despite the trade-off in insulation efficiency. The prepared sample with yeast exhibited a characteristic U-value that indicates the biological mechanism of the yeast, which allows a gas release of carbon dioxide produced during fermentation, resulting in finely distributed micro-porosity within the sample. The uniform and stable pores formed trap air effectively, reducing heat transfer. Such results align well with prior research where yeast was utilized as a bio-foaming agent to create lightweight construction materials with tightly controlled pore structures^99^.

The sample that used baking powder showed lower thermal performance than the others. Baking powder is a strong chemical foaming agent that releases CO_2_ through a reaction between its acidic and basic components during processing^100^. At lower temperatures, this reaction creates pores in the material. Still, at higher temperatures, sodium carbonate may cause the particles to soften and compact more tightly, reducing the likelihood of pore formation. This observation is confirmed with SEM, as well as the apparent porosity results.

Based on the previous discussion, the maximum U-value recorded reached 0.388 W/m^2^K. This seems to relate to the BP sample that produced fewer pores, thus showing decreased insulation capabilities that emphasize optimizing foaming agent content and particle sizes for reducing heat transfer pathways. The analysis of U-values between 0.327 and 0.388 W/m^2^K demonstrates how natural foaming agents serve as essential tools for shaping recycled waste-derived building materials while meeting environmental needs and thermal performance standards. The biological action of yeast produces superior insulating cellular structures, which establishes it as a highly effective foaming agent. The chemical foaming agent, baking powder, presents functional benefits alongside moderate thermal resistance, and agricultural residues provide cost-effective, eco-friendly solutions that result in diminished thermal performance.

**Table 5:** The descriptive analysis of the U-values of the prepared samples at 1250 °C.

Analysis	Temp.	*N*	Mean	SD	SEM	Test statics		
						T-static	DF	Prop>ǀtǀ
U-values	1250 °C	6	0.35	0.023	0.009	37.38	5	2.57 × 10^− 7^

Table 5 represents the statistical analysis of the U-values for the samples sintered at 1250 °C, indicating a mean value of 0.35 with a standard deviation of 0.023 and a standard error of the mean (SEM) of 0.009, reflecting a relatively small dispersion among the measured samples and suggesting good consistency in the thermal performance of the prepared material. The low standard deviation demonstrates that the U-values are closely distributed around the mean, indicating uniform microstructural characteristics among the specimens produced under the same sintering conditions. The *t*-test results yielded a t-statistic of 37.38 with 5 degrees of freedom and an extremely small p-value of 2.57 × 10^− 7^, which is far below the significance level (α = 0.05). This very high *t*-value, combined with the extremely low probability value, indicates that the measured U-values are statistically significant and highly reliable. Such stability in the U-value suggests that the thermal transfer properties of the samples are strongly controlled by the microstructure formed during sintering at 1250 °C, where the distribution of pores and solid phases remains relatively homogeneous. The low variability further implies that the preparation method provides reproducible thermal insulation characteristics, which is an important factor for materials intended for thermal management or insulation applications.

### FTIR absorption spectra

**Fig. 10:**
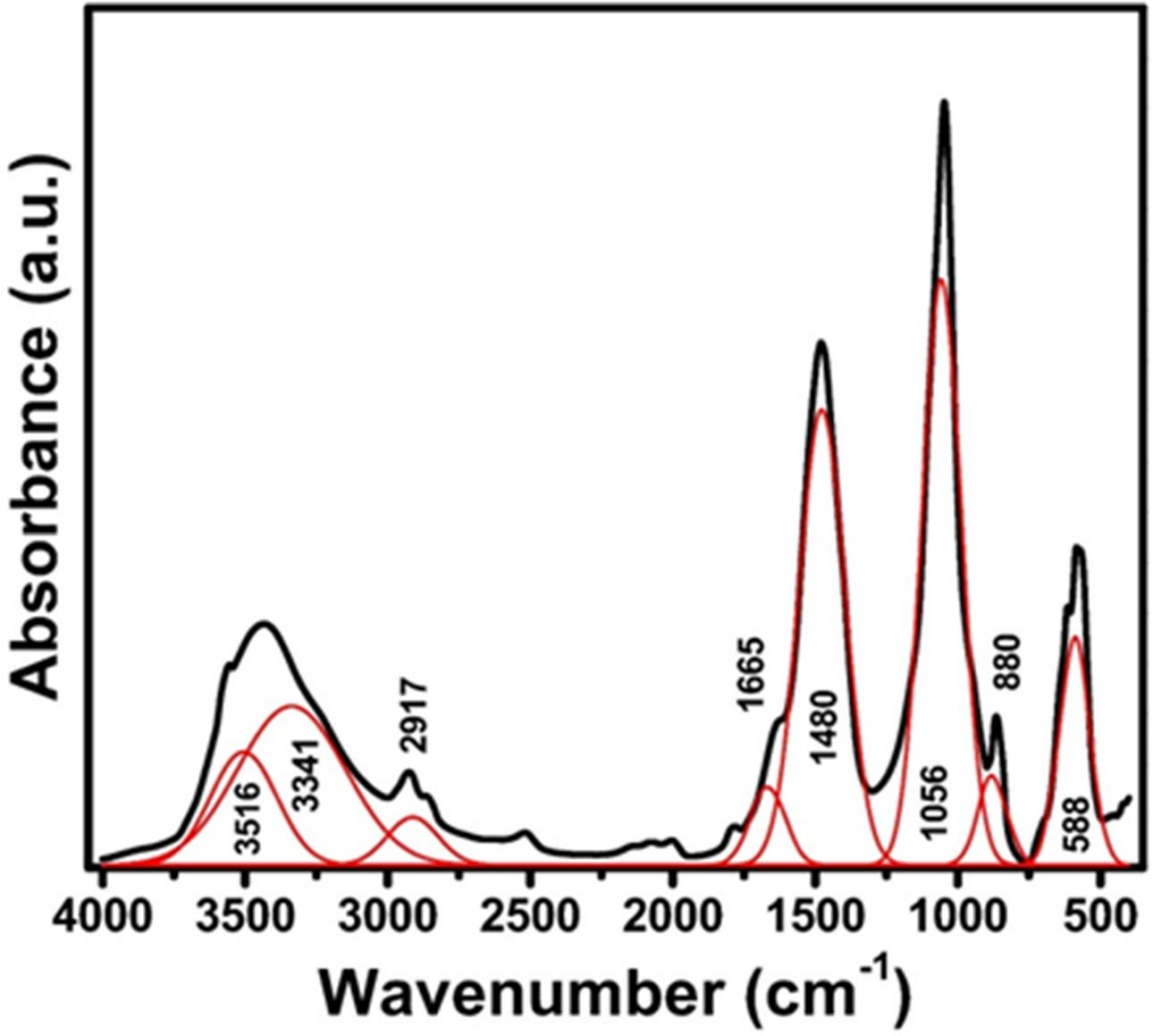
FTIR absorption spectra of the fired blank sample.

Figure 10 depicts the FTIR spectrum of the foamed material derived from crushed construction waste (sample containing starch), highlighting the leading absorption bands at 588, 880, 1056, 1480, 1665, 3341, 3516, and 3917 cm^− 1^. These bands allow identification of the chemical composition, existing functional groups, rearrangements of the basic structural units, and even some phase changes in the ceramic derived from construction waste, as detailed below^101–110^:

(i) The absorption band at 588 cm^-1^ is typically attributed to the stretching vibrations of metal-oxygen (M-O) bonds, particularly the Si-O-Si bending, as well as other common metal oxides that might be present in construction waste materials, which are the most common, such as calcium, aluminium, or iron oxides. With this band, it is possible to identify the occurrence of a silicate network or a combination of metal oxides that have been formed during the process of foaming and sintering, confirming the presence of a ceramic matrix. The appearance of this band is proof of the formation of the stable oxide linkages, which are the basic structural support of the foamed ceramic.
(ii) The infrared absorption band at 880 cm^-1^ is usually associated with the bending vibrations of carbonate groups (CO_3_^2-^). The mentioned features imply that certain calcium carbonate phases (such as calcite or aragonite) are present in the prepared material matrix; hence, they are the most likely to come from the rocks or cement additives in the waste. Moreover, the thermal treatment has been inefficient in breaking down carbonates. Therefore, some original minerals from the construction debris still have not reacted or have been combined with the ceramic structure.
(iii) The sharp, highly intense spectral band observed at 1056 cm^-1^ corresponds to the asymmetric stretching vibrations of silicate bridge bonds in Si-O-Si structures. This characteristic peak represents the siloxane network, which forms the main structure of the ceramic framework. The band confirms that the ceramic mainly consists of silicate materials originating from crushed concrete, brick, and glass waste components. This vibrational mode indicates polymerization and network formation within the silicate glassy phase, strengthening the foamed ceramic’s mechanical properties.
(iv) The absorption band around 1480 cm^-1^ indicates the asymmetric stretching vibrations of the carbonate group (CO_3_^2-^), with the primary source being the calcite impurities that co-exist in the waste used to produce building materials. Such a peak complements the earlier identification at 880 cm^-1^, suggesting that the occurrence of the carbonates is either physically trapped or they have been combined with the ceramic matrix. Besides that, these carbonates may also be the reason for the changes in the material’s thermal properties, as well as the gradual porosity formed during the foam process; therefore, they would be considered as agents of the foam by their decomposition.
(v) The band at 1665 cm^-1^ is typically identified as the bending vibration of water molecules that have been adsorbed on the surface or hydroxyl groups (H-O-H bending). Such a peak suggests that the ceramic surface, and maybe the pores inside, have water physically adsorbed or shallow hydroxyl groups resulting from the hydration stages of construction waste before sintering or water adsorbed from the atmosphere after sample preparation. Adsorbed water can change the performance of the foamed ceramic with respect to its thermal and mechanical behaviour.
(vi) The broad absorption bands at 3341 and 3516 cm^-1^ are related to the stretching vibrations of hydroxyl groups (OH^-^) and water molecules. Usually, this range shows O- H stretching modes for surface-bound or structural water and hydroxyl groups, which are the main source of silicate minerals or hydrated phases.
(vii) The band at 3917 cm^-1^ indicates unusual features that may be attributed to hydroxyl overtone combination bands or high-energy vibrations of water molecules in porous ceramics. It is also possible that this band arises from silanol groups on the surface, which is in line with the previous results of bands related to hydroxyl and water, as the hydrogen bonds are formed. The changed band in the spectrum and moisture-related species are the representatives of the ceramic surface chemistry and its pore surface reactivity.

## Conclusion

The study introduces a practical and environmentally responsible approach to producing porous ceramic materials by recycling waste concrete and combining it with a starch-based strengthening method and various pore-forming agents, including flour, yeast, baking powder, sawdust, and wheat straw. In this approach, starch plays a dual role: it binds the ceramic particles together and also acts as a source for gas generation, which helps create the porous structure during the formation of the green bodies. These samples were then sintered at temperatures of 1200 °C, 1250 °C, and 1300 °C. A comprehensive set of characterization techniques including TGA, measurements of apparent porosity, bulk density, compressive strength, microstructural observation, phase analysis, FTIR and thermal transmittance was used to evaluate their performance. The results showed that, except for samples containing baking powder, increasing the firing temperature generally reduced porosity while improving density and mechanical strength. Flour-based samples developed a highly porous structure, reaching porosity values of 56% and 39%, bulk densities of 1.27 and 1.64 g/cm³, and compressive strengths of 0.55 and 1.47 MPa at 1250 °C and 1300 °C, respectively, with a thermal transmittance of 0.327 W/m²K at 1250 °C. Sawdust-containing samples, on the other hand, showed the best mechanical performance, with compressive strengths of 1.54 MPa at 1250 °C and 5.23 MPa at 1300 °C, while maintaining porosity levels of about 55% and 38% and thermal transmittance values up to 0.336 W/m²K. Overall, the ceramics exhibited low thermal conductivity after firing at 1250 °C, highlighting their suitability for thermal insulation applications. Although baking powder offered certain functional advantages with moderate thermal resistance, agricultural waste materials provided a cost-effective and environmentally friendly alternative, even if with slightly lower thermal performance. Altogether, this method presents an efficient way to replace expensive polymer additives with recycled concrete waste, resulting in porous ceramic materials with promising applications in gas burners, membranes, filtration systems, and refractory insulation.

## Data Availability

The datasets used and/or analysed during the current study available from the corresponding author on reasonable request.
